# Low protein diet supplemented with ketoacids on muscle wasting in chronic kidney disease: A clinical trial

**DOI:** 10.3389/fmed.2022.949108

**Published:** 2022-08-30

**Authors:** Yueyue Zhang, Lijie Gu, Ling Wang, Shu Rong, Weijie Yuan

**Affiliations:** Division of Nephrology, Shanghai General Hospital, Shanghai Jiao Tong University School of Medicine, Shanghai, China

**Keywords:** chronic kidney disease, low protein diet, ketoacids, skeletal muscle wasting, body composition analysis

## Abstract

**Aim:**

Nutrition is an important part of the care of patients with chronic kidney disease (CKD). However, there is limited clinical research on the skeletal muscle nutrition of patients with CKD. We carried out this study to find out whether a low-protein diet supplemented with ketoacids (LPD + KA) could improve muscle wasting in patients with CKD.

**Methods:**

Patients were enrolled in this non-blind, parallel-group, randomized trial assessing the nutritional status of CKD, randomly assigned to either the LPD + KA group or conventional LPD group. Blood samples such as Hemoglobin, Cystatin C, Creatinine, BUN, Albumin, Pre- Albumin, Glycerin Trilaurate, and Cholesterol were measured at baseline and every 3 months. The parameters of skeletal muscle and other body composition were assessed before and after dietary intervention for 12 months.

**Results:**

A total of 58 patients with CKD completed the study and were available for further analysis. The hemoglobin and albumin were observed to be markedly improved in the LPD + KA group during the follow-up as compared to baseline. Body mass index and total body water index of both groups were increased upon follow-up but the increase in the LPD + KA group was comparatively higher. Moreover, an increase in body fat%, skeletal muscle mass index, and appendicular skeletal muscle mass index was observed in both groups between baseline and follow-up, but it was statistically insignificant.

**Conclusion:**

This study did not find a significant improvement of KAs on muscle wasting, and a long time or more indices study may need to find the effects of the LPD + KA diets.

**Clinical trial registration:**

[www.ClinicalTrials.gov], identifier [NCT02568020].

## Introduction

Chronic kidney disease (CKD) makes complex metabolic processes alter and affect muscular homeostasis, leading to a loss of muscle mass and, ultimately leading to muscle atrophy (1, 2). There are about 16–54% of patients with CKD are dystrophic (3). The syndromic uremic dystrophy has been nominated as protein-energy wasting (PEW) (4), is the main characteristic of PEW, indicating the simultaneous losses in protein and energy storage (5), increasing as CKD progresses, and ultimately increasing the risk of mortality (6, 7).

Nutrition is an important part of the care of patients with CKD. For decades, protein restriction has been used to improve complications such as abnormal glucose metabolism and hypertension in patients with CKD and protect remnant kidneys’ function. As in long-term protein restriction, decreased amino-acid supply may lead to decreased protein synthesis and malnutrition, we often prescribed a low-protein diet (LPD) together with ketoacids (KAs), a nitrogen-free substitution for the essential amino acids, to patients with advanced CKD (8, 9).

Ketoacids supplementation may be protective against muscle atrophic in animal models (10–13). In 5/6th nephrectomy rats, LPD supplemented with ketoacids (LPD + KAs) was able to inhibit the activation of the ubiquitin-proteasome system and protect skeletal muscle from atrophy and oxidative damage when compared with LPD alone. KAs can prevent the decreased activity of the mitochondrial electron transport chain complexes and increase mitochondrial respiration (10, 12, 13). LPD + KAs decreased muscle autophagy markers, but no difference in inflammation in CKD skeletal muscle (11).

Although the reviewed evidence seems to suggest that KAs supplementation can be expected to bring positive results, there is limited clinical research on the skeletal muscle nutrition of patients with CKD. We, therefore, carried out this study, and divided subjects into the LPD group or LPD + KAs group, following up and testing their blood assay and body composition analysis.

## Materials and methods

### Study population

Between 26 October 2016 and 13 May 2020, subjects were enrolled from the Nephrology department of Shanghai General Hospital, Shanghai Jiao Tong University School of Medicine, Shanghai, China, as part of this non-blind, parallel-group, randomized trial assessing the nutritional status of CKD (This trial was registered at www.ClinicalTrials.gov as NCT02568020). All individuals came from the outpatient clinics of Shanghai General Hospital. Once identified, they continued routine medical care in their clinics and had an investigative follow-up. Only those who finished the follow-up were further analyzed. Demographics of the patients were obtained from medical records or patient interviews.

Inclusion criteria: patients agree to participate in this study; age ≥ 18 years and < 70 years; renal function measured with creatinine clearance [by Modification of Diet in Renal Disease equation (10)] < 60 and > 15 ml/min (3 monthly consecutive measurements); at least 6 months of follow up at our clinic before recruitment and haven’t received any diet intervention.

Exclusion criteria: pregnant patients; diabetes; heart or liver failure; a recent myocardial infarction (in the last 12 months); long-term immobilization; chronic respiratory failure; cancer; any pharmacological treatment that could modify muscle structure or function such as glucocorticoids or insulin; contraindications of Ketosteril, such as hypersensitivity to the active substances or to any of the excipients, hypercalcemia, disturbed amino acid metabolism to the study protocol. Furthermore, patients who were ≥ 150 kg body weight, had a physical disability, or have metal implants were excluded to make sure the prerequisites of body composition were monitored.

All protocols were approved by the Institutional Review Board of Shanghai Jiao Tong University Affiliated Shanghai General Hospital. Written informed consent was obtained from all patients or their closest family members. This research was conducted in adherence to the principles of the Helsinki Declaration of 1975 as revised in October 2013.

### Treatment regimen

Patients were randomly assigned to either conventional LPD (LPD group, 30 patients) or LPD + KAs (LPD + KAs group, 30 patients). All patients were treated with an LPD containing 0.6 g protein/kg body weight per day and 120–125 kJ/kg body weight per day. Besides, the LPD + KAs group will be supplemented with keto-amino acids (Ketosteril^®^, Fresenius Kabi) at a dosage of one tablet/5 kg ideal body weight/day, divided into three doses taken during meals.

### Follow-up

We obtained the subjects’ dietary and medication intake through questionnaires every 3 months, operated a computer-based nutritional evaluation with diet software, and reminded the patients to have a 0.6 g protein/kg protein diet.

**FIGURE 1:**
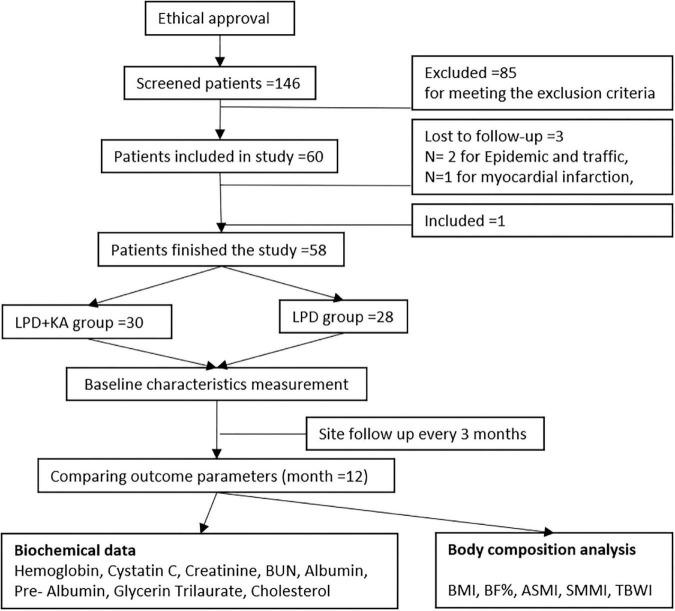
Study methodological flow diagram. KAs, ketoacids; LPD, low-protein diet; BMI, body mass index; BF, body fat; SMMI, Skeletal Muscle Mass Index; ASMI, appendicular skeletal muscle mass index; TBWI, total body water index.

The patients were followed up for 1 year. During follow-up, three patients were lost to follow-up, in which two cases were for epidemic and traffic reasons, with one case was for myocardial infarction, which were thought to have nothing to do with medication. Then one case was included to make up the number and finally completed the study. The methodological process of the current study is described in Figure 1.

### Biochemical data

Blood samples were collected at baseline and every 3 months. All the patients were told to fast overnight and were collected 5 ml of blood the next morning between 7:00 and 7:30 a.m. Hemoglobin, Cystatin C, Creatinine, BUN, Albumin, Pre-Albumin, Glycerin Trilaurate, and Cholesterol were measured at local sites by using standard techniques.

### Body composition analysis

The parameters of body composition were assessed at baseline and at the end of follow-up. Body composition was determined through the Donghuayuan body composition monitor (DBA-550). It works on the principle of bioelectrical impedance. Variables such as sex, age, and height were input into the monitor. Patients were asked to stand barefoot on the foot electrodes of the measurement platform, with knees and back upright and straight without moving, then hold the grip electrodes firmly with their hands and extend their arms straight at a 45° angle to their body. As the measurement was completed, body mass index (BMI), percent body fat (BF%), skeletal muscle mass, in intracellular water (ICW), extracellular water (ECW), and total body water (TBW) appeared on the report. Appendicular skeletal muscle mass index (ASMI), Skeletal Muscle Mass Index (SMMI), and total body water index (TBWI) were calculated. ASMI was calculated as the sum of lean mass for the arms and legs (kilograms)/height^2^ (meters^2^) (14). Accordingly, SMMI was calculated as the sum of lean mass for the body, arms, and legs (kilograms)/height^2^ (meters^2^), and TBWI was calculated as the total body water (kilograms)/height^2^ (meters^2^).

### Study endpoint

The endpoint is the arrival point or the patient is converted to dialysis. The initiation of chronic dialysis was obtained through participant self-reporting during the follow-up period and was verified by the clinical and hospital records at the local site.

The primary efficacy variable will be ASMI. Secondary efficacy variables will be BMI, BF%, and SMMI.

Safety variables will be serum creatinine.

### Statistical analysis

The normally distributed measurement data were expressed as mean ± standard deviation (*SD*), the comparison between two groups was performed by *t*-test, and the comparison among multiple groups was performed by analysis of variance. The differences between the same periods comparing the two groups were analyzed using an independent Student’s *t*-test. The same group between baseline and 12 months was compared by Paired Student’s *t*-test. The sample size calculation assumes that the primary variable is the percentage change from baseline ASMI. With 30 patients per group, a one-sided *t*-test at a significance level of 2.5% has a power of 80% to detect an improvement of 7.36% in the LPD + KA group vs. the LPD group. *P* < 0.05 was considered statistically significant, and SPSS 20.0 statistical software was used for analysis.

## Results

A total of 58 patients with CKD, 28 in the LPD group and 30 in the LPD + KAs group, completed the study and were available for further analysis. Characteristics and blood assays of the study participants at baseline and at the end of 1-year follow-up are shown in Tables 1, 2, and no participant converted to dialysis during the follow-up.

**TABLE 1:** Patient demographics and characteristics.

Characteristic	LPD + KAs group	LPD group
Gender (male%)	21/30	18/28
Ages	56.16 ± 12.39	54.39 ± 8.18
**Cause of chronic kidney disease**		
Hypertension	1/30	1/28
Glomerular disease	1/30	0/28
Presumed chronic glomerular disease	20/30	20/28
IgA Nephrology	1/30	0/28
Others	7/30	7/28
**Complication and medical history**		
Hypertension	18/30	20/28
Coronary artery disease	5/30	4/28
Hyperuricemia	10/30	10/28

KAs, ketoacids; LPD, low-protein diet.

**TABLE 2:** Biochemical data of study participants at baseline and at the end of follow-up.

	Baseline			1 year-follow up			Pair-comparison-LPD + KAs group	Pair-comparison-LPD group
	LPD + KAs group	LPD group	Sig	LPD + KAs group	LPD group	Sig	Sig	Sig
Hemoglobin (g/l)	121.99 ± 21.41	126.21 ± 16.94	0.395	133.41 ± 28.64	127.44 ± 23.1	0.386	0.001	0.685
Cystatin C(mg/l)	1.88 ± 0.67	1.72 ± 0.49	0.317	2.75 ± 1.3	2.08 ± 0.7	0.171	0.305	0.351
Creatinine (μmol/l)	181.17 ± 100.85	139.5 ± 62.84	0.058	220.76 ± 199.31	165.35 ± 155.36	0.230	0.107	0.316
Blood urea nitrogen (mmol/l)	10.46 ± 5.27	8.9 ± 3.12	0.167	12.36 ± 9.58	10.94 ± 7.78	0.533	0.146	0.089
Albumin (g/l)	40.77 ± 4.44	39.83 ± 3.07	0.340	42.37 ± 4.43	41 ± 4.54	0.246	0.005	0.161
Pre-Albumin (mg/l)	297.87 ± 109.58	257.9 ± 65.56	0.097	298.69 ± 66.01	299.19 ± 87.6	0.981	0.901	0.029
Glycerin Trilaurate (mmol/l)	1.89 ± 1.08	1.63 ± 0.72	0.290	2.2 ± 1.95	1.76 ± 0.78	0.454	0.367	0.445
Cholesterol (mmol/l)	4.49 ± 0.77	4.74 ± 1.28	0.371	4.74 ± 0.91	4.68 ± 1.34	0.822	0.093	0.566

KAs, ketoacids; LPD, low-protein diet.

**FIGURE 2:**
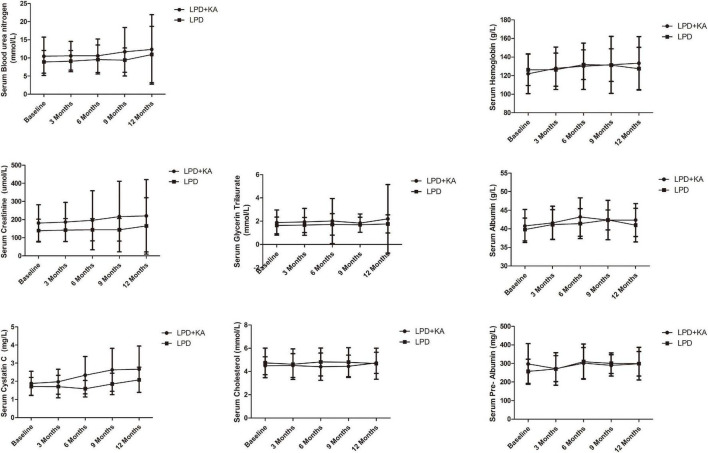
Biochemical data of study participants at baseline and every 3 months during follow-up. KA, ketoacids; LPD, low-protein diet.

It could be seen from Table 2 and Figure 2 that, a progression was observed in cystatin C, creatinine, and blood urea nitrogen between baseline and 1-year follow-up in both groups but it was statistically insignificant (*p* > 0.05). It could be seen that, in LPD group, pre-Albumin was markedly improved (*p* < 0.05), hemoglobin, albumin, and cholesterol was improved statistically insignificant (*p* > 0.05), while glycerin trilaurate progressed but statistically insignificant (*p* > 0.05). In LPD + KA group, the hemoglobin and albumin were observed to be markedly improved at the end of 1-year followed up as compared to the baseline (*p* < 0.05). Although an improvement was beheld in pre-Albumin, glycerin trilaurate, cholesterol of LPD + KA group this change was statistically insignificant (Table 2 and Figure 2, *p* > 0.05).

Body composition analysis at baseline and the end of 1-year follow-up is shown in Table 3. BMI and TBWI in the LPD + KA group were comparatively higher and increased upon follow-up (*p* < 0.05). Moreover, an increase in BF%, SMMI, and ASMI were observed in both groups between baseline and follow-up, but it was statistically insignificant (*p* > 0.05).

**TABLE 3:** Body composition analysis of study participants at baseline and at the end follow up.

	Baseline			1 year-follow up			Pair-comparison-LPD + KAs group	Pair-comparison-LPD group
	LPD + KAs group	LPD group	Sig	LPD + KAs group	LPD group	Sig	Sig	Sig
BMI (kg/m^2^)	23.92 ± 3.67	24.3 ± 3.65	0.683	24.52 ± 4.05	24.69 ± 4.19	0.873	0.045	0.239
BF% (%)	25.03 ± 8.29	27.36 ± 7.68	0.255	25.06 ± 8.59	27.45 ± 8.1	0.265	0.894	0.755
SMMI (kg/m^2^)	15.23 ± 2.17	14.9 ± 1.64	0.506	15.31 ± 2.42	14.98 ± 1.96	0.565	0.633	0.614
ASMI (kg/m^2^)	7.42 ± 1.11	7.12 ± 0.91	0.263	7.50 ± 1.40	7.25 ± 1.23	0.453	0.513	0.313
TBWI (kg/m^2^)	13 ± 1.76	12.77 ± 1.3	0.561	13.31 ± 1.93	13.02 ± 1.66	0.530	0.028	0.127

KAs, ketoacids; LPD, low-protein diet; BMI, body mass index; BF, body fat; SMMI, Skeletal Muscle Mass Index; ASMI, appendicular skeletal muscle mass index.

## Discussion

Nutrition is a key component of care during CKD. There were findings suggesting that patients with CKD inhibiting protein intake can obtain the adaptation of muscle protein metabolism through the combined response to protein degradation and the recycling efficiency of protein breakdown amino acids. KAs supplementation has been proposed to reduce the risk of nutritional disorders of a very low-protein diet (VLPD), and VLPD + KAs were included as part of the clinical recommendations for patients with CKD in KDOQI Clinical Practice Guideline for Nutrition in CKD: 2020 Update (15): for CKD adults without diabetes, with an eGFR < 20 mL/min/1.73 m^2^ (before dialysis), a VLPD with 0.28 to 0.43 g protein/kg plus KAs added to meet protein requirements were recommended.

In this study, a progression was observed in renal function (cystatin C, creatinine, blood urea nitrogen) between baseline and 1-year follow-up in both groups but it was statistically insignificant. This may be because reducing protein intake can delay hyalinosis, and reduce proteinuria and glomerular hyperfiltration (15). In the LPD group, pre-Albumin was markedly improved, but hemoglobin, albumin, and cholesterol improved statistically insignificant, while glycerin trilaurate progressed but was statistically insignificant. While in the LPD + KA group, hemoglobin and albumin were markedly improved, similar to the previous reference (16), but the improvement of pre-Albumin, glycerin trilaurate, and cholesterol were statistically insignificant. As in a *post hoc* analysis of the MDRD Study (17), the authors compared the various outcomes of LPD and VLPD + KAs related to nutritional status, and found the safety of dietary protein restriction over 2–3 years in patients with moderate to advanced CKD. Serum albumin levels were elevated, while serum transferrin levels and urine creatinine excretion were decreased in LPD and VLPD groups. Also, the review by Koppe et al. showed that VLPD + KAs appears to reduce the production of uremic toxins, acidosis, phosphorous, and possible sodium intake while providing an adequate calcium intake (18).

As to body composition in this study, BMI and TBWI in the LPD + KA group were significantly increased, but the increase of BF%, SMMI, and ASMI was not statistically significant, indicating that the increase in BMI may be related to the change in TBWI. According to analysis in the MDRD study (17), body weight, BF%, and arm muscle area were decreased in LPD and VLPD groups. As reported in a longitudinal study looking at body composition, a VLPD + KA caused a small decline in lean body mass, accompanied by an increase in fat mass, mainly during the first 3 months (16). These parameters were subsequently stabilized and even improved slightly thereafter.

There were other short-term studies that did not show a significant effect of LPDs and VLPDs + KAs on nutritional parameters. And there are also some studies that observed the small anthropometric measurement declines which may be for the prolonged use of LPDs and VLPDs + KAs in routine practice and the adverse effect of end-stage kidney disease. That is why physicians who prescribe LPDs must regularly monitor patients for their protein and energy intake, body weight, and nutritional status. Although this diet is not associated with wasting in carefully monitored studies, attention should focus on energy intake, which may decrease over time and lead to weight loss and consumption.

There were many limitations in this study, although there was a significant change in serum hemoglobin and albumin, neither of them was a direct indicator of muscle wasting. As for the completeness of the low-protein diet or the daily protein intake, it is not guaranteed. But it was documented that medication adherence is strongly associated with patient medication knowledge (19, 20), and it can be speculated that the same is true for dietary adherence. As to the study, we may need more time and more indices to find the effects of the LPD + KA diets in the following studies, and more research is needed to examine the effectiveness of KAs, especially on muscle wasting.

## Data availability statement

The original contributions presented in this study are included in the article/supplementary material, further inquiries can be directed to the corresponding author.

## Ethics statement

The studies involving human participants were reviewed and approved by the Institutional Review Board of Shanghai Jiao Tong University Affiliated Shanghai General Hospital. The patients/participants provided their written informed consent to participate in this study.

## Author contributions

YYZ explored the data and wrote the manuscript. LJG and LW collected the data. SR reviewed this manuscript. WJY designed this study and reviewed this manuscript. All authors contributed to the article and approved the submitted version.

## Abbreviations

ASMI: appendicular skeletal muscle mass index
BF: body fat
BMI: body mass index
CKD: Chronic kidney disease
KAs: ketoacids
LPD: low-protein diet
PEW: protein-energy wasting
SMMI: Skeletal Muscle Mass Index
TBWI: total body water index.
